# Development of a High-Performance Thin-Layer Chromatography Method for the Quantification of Alkyl Glycerolipids and Alkenyl Glycerolipids from Shark and Chimera Oils and Tissues

**DOI:** 10.3390/md20040270

**Published:** 2022-04-18

**Authors:** Marion Papin, Cyrille Guimaraes, Benoit Pierre-Aue, Delphine Fontaine, Jeoffrey Pardessus, Hélène Couthon, Gaëlle Fromont, Karine Mahéo, Aurélie Chantôme, Christophe Vandier, Michelle Pinault

**Affiliations:** 1 Nutrition, Croissance, Cancer (N2C) UMR 1069, University of Tours, INSERM, 37000 Tours, France; marion.papin@etu.univ-tours.fr (M.P.); cyrille.guimaraes@gmail.com (C.G.); benoit.paue45@gmail.com (B.P.-A.); delfontaine12@gmail.com (D.F.); gaelle.fromont-hankard@univ-tours.fr (G.F.); karine.maheo@univ-tours.fr (K.M.); aurelie.chantome@univ-tours.fr (A.C.); michelle.pinault@univ-tours.fr (M.P.); 2 Centre d’Étude des Pathologies Respiratoires (CEPR)-U1100, University of Tours, INSERM, 37000 Tours, France; jeoffrey.pardessus@univ-tours.fr; 3 Laboratoire Chimie Electrochimie Moléculaires et Chimie Analytique (CEMCA) UMR 6521, University of Brest, CNRS, 29238 Brest, France; helene.couthon@univ-brest.fr

**Keywords:** ether lipids, chromatography, oils, tissues

## Abstract

Ether lipids are composed of alkyl lipids with an ether bond at the *sn-1* position of a glycerol backbone and alkenyl lipids, which possess a vinyl ether bond at the *sn-1* position of the glycerol. These ether glycerolipids are present either as polar glycerophospholipids or neutral glycerolipids. Before studying the biological role of molecular species of ether glycerolipids, there is a need to separate and quantify total alkyl and alkenyl glycerolipids from biological samples in order to determine any variation depending on tissue or physiopathological conditions. Here, we detail the development of the first high-performance thin-layer chromatography method for the quantification of total alkyl and alkenyl glycerolipids thanks to the separation of their corresponding alkyl and alkenyl glycerols. This method starts with a reduction of all lipids after extraction, resulting in the reduction of neutral and polar ether glycerolipids into alkyl and alkenyl glycerols, followed by an appropriate purification and, finally, the linearly ascending development of alkyl and alkenyl glycerols on high-performance thin-layer chromatography plates, staining, carbonization and densitometric analysis. Calibration curves were obtained with commercial alkyl and alkenyl glycerol standards, enabling the quantification of alkyl and alkenyl glycerols in samples and thus directly obtaining the quantity of alkyl and alkenyl lipids present in the samples. Interestingly, we found a differential quantity of these lipids in shark liver oil compared to chimera. We quantified alkyl and alkenyl glycerolipids in periprostatic adipose tissues from human prostate cancer and showed the feasibility of this method in other biological matrices (muscle, tumor).

## 1. Introduction

**Figure 1:**
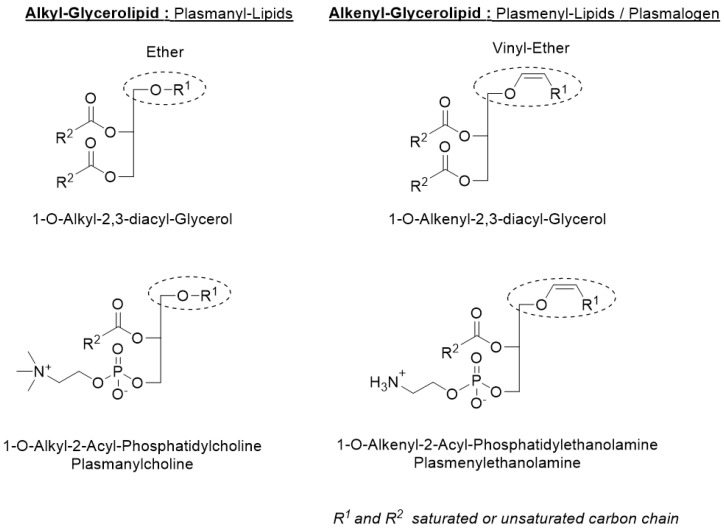
Chemical structures of ether lipids. Ether lipids are composed of neutral glycerolipids and polar glycerophospholipids. Alkyl glycerolipids have an alkyl chain attached by an ether bond at the *sn-1* position; they are referred as plasmanyl lipids. The *sn-2* position generally has an ester-linked acyl chain. Some ether lipids (alkenyl glycerolipids) have a cis double bond, vinyl ether adjacent to the ether linkage and are also named plasmenyl lipids or plasmalogens. For glycerophospholipids, the *sn-3* position has a polar head group, generally choline for plasmanyl lipids and ethanolamine for plasmenyl lipids (except in the heart, which has a significant proportion of plasmenylcholine lipids).

Ether lipids, which are composed of neutral glycerolipids and polar glycerophospholipids in animals, are a family of lipids divided into two subfamilies: alkyl lipids (also known as plasmanyl lipids), with an ether bond at the *sn-1* position of the glycerol backbone; and alkenyl lipids (also known as plasmenyl lipids or plasmalogens), which possess a vinyl ether bond at the *sn-1* position of the glycerol (Figure 1) (for reviews, see [1,2]). Ether lipids are mainly present either as neutral glycerolipids or polar glycerophospholipids, depending on the tissue. For example, it has been shown that the heart is one of the organs that contains the most glycerophospholipids in contrast to adipose tissue, whereas the opposite is true for neutral glycerolipids [3]. It has been reported that human neutral glycerolipids contain a higher content of alkyl lipids compared to alkenyl lipids, the latter generally predominate in glycerophospholipids [4,5]. In addition, alkyl lipids are generally more prevalent in the choline than ethanolamine glycerophospholipids, in contrast to what is observed for alkenyl lipids (Figure 1, [6,7]). Alkenyl lipids have mostly either a C16:0, C18:0, or C18:1 fatty alcohol (with CX:Y, X the number of carbon atoms in the chain and Y the number of unsaturations) in the *sn-1* position of the glycerol and are particularly enriched in n-3 and n-6 polyunsaturated fatty acids, such as docosahexaenoic acid (22:6n-3) or arachidonic acid (20:4n-6), in the *sn-2* position, which makes them a reservoir of many second messengers and metabolites, especially in the arachidonic acid metabolic pathway [8,9]. Ether lipids are ubiquitous in animals, although their distribution and composition vary greatly depending on the species and organs studied [2]. In mammals, ether lipids are generally considered to represent 20% of total phospholipids [10]; however, organs such as the heart, brain, spleen, testes, kidneys, skeletal muscle, lungs, some immune cells, erythrocytes and bone marrow [2,11,12] have high amounts of ether lipids (up to more than 50% of total phosphatidylethanolamines in the brain and heart) [8]. These lipids are also found in breast milk [13,14] and are thought to help maintain brown adipose tissue in breastfed infants, thereby reducing the development of obesity in young children [15]. Endogenous alkyl glycerolipids were found in the liver oil of various chondrichthyans [2], and we recently reported the following distribution in shark and chimera liver oils by gas chromatography with C14:0 (20–24%), C16:0 (42–54%) and C18:1 (6–16%) and, to a lesser extent, (0.2–2%) for each of the following: C16:1, C17:0, C18:0 and C20:0 [16].

A well-known problem in ether lipid analysis is the quantification and separation of alkyl and alkenyl lipids because of incorrect annotations of species, the restricted selection of ether lipid standards and incomplete databases used for mass spectrometry experiments. Recently, Koch et al. used the newly discovered TMEM189/PEDS1 (Transmembrane Protein 189, also named plasmanylethanolamine desaturase 1) enzyme to develop a new mass spectrometry method that permits differentiation between alkenyl lipids and other ether lipids [17,18]. Here, we develop a simple and quantitative high-performance thin-layer chromatography method (HPTLC) for the quantification of alkyl lipids and alkenyl lipids thanks to their separation in their ether glycerol form.

The aim of this study was to develop a simple method to assess the total quantity of alkyl and alkenyl glycerolipids separately because we believe quantifying these two forms of ether lipids is important. This was achieved by developing a procedure consisting of a first step of reduction of all lipids, resulting in the reduction of alkyl and alkenyl glycerolipids to alkyl and alkenyl glycerols, whereas their ester analogues are reduced to glycerol. This procedure is followed by a step of separation by HPTLC of alkyl and alkenyl glycerols, followed by a step of densitometric quantification of alkyl and alkenyl glycerols, directly obtaining quantities of alkyl and alkenyl glycerolipids, respectively. The HPTLC method was validated following the guidelines provided by the International Conference on Harmonization (ICH) [19] to assess the selectivity, linearity, sensitivity, accuracy, repeatability and precision of the method. Finally, this method was tested on various biological matrices to assess its feasibility for biomedical research.

## 2. Results and Discussion

### 2.1. Principle of the Method

**Figure 2:**
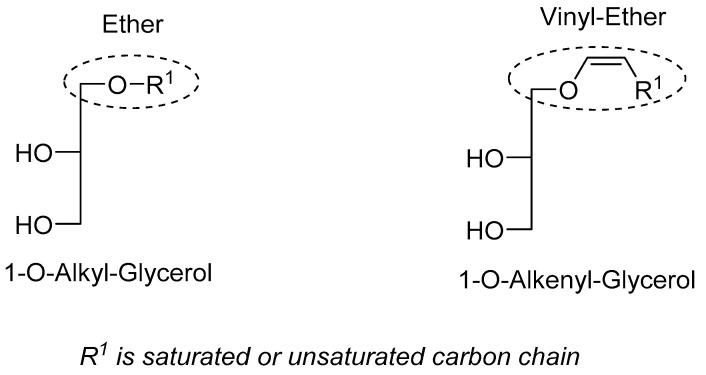
Structures of the ether glycerols obtained after reduction with Vitride^®^. Vitride^®^ reduces ester and phosphate bonds to alcohol but not ether or vinyl ether bonds. Ether lipids (bearing on the *sn-1* position an ether or vinyl ether bond; on *sn-2*, an ester bond; and on *sn-3*, an ester or phosphate bond) are reduced to ether glycerols (1-O-alkyl glycerol for alkyl lipids and 1-O-alkenyl glycerol for alkenyl lipids, referred to as alkyl glycerol and alkenyl glycerol, respectively). Ester lipids are reduced to glycerol and fatty acids.

The proposed method was adapted from the TLC method described by Renkonen et al. [20]. Because we aimed to assess the total quantity of alkyl and alkenyl glycerolipids in both their neutral and polar forms and because their polarities are not different enough when bearing an acyl and/or phosphate bond in the *sn-2* and *sn-3* position, this method starts with a reduction of all alkyl and alkenyl glycerolipids to alkyl and alkenyl glycerols, respectively (Figure 2; see Section 3.8.2). This reduction step fully reduces the neutral and polar ester analogues of ether glycerolipids to glycerols (Figure S6), which have a different polarity and no longer interfere with the separation of alkyl and alkenyl glycerols. Then, by separating alkyl and alkenyl glycerols thanks to HPTLC and quantifying them by densitometric analysis after carbonization, we directly quantify the total amount of alkyl and alkenyl glycerolipids separately.

### 2.2. HPTLC Method Validation Using Standards

The HPTLC method (see Section 3) for the separation of alkyl and alkenyl glycerols was first optimized and validated using commercial ether glycerol standards. The mobile phase was optimized (Figure S1, Supplementary), enabling a clear separation between the ether glycerol standards and proving the selectivity of the proposed method regarding alkyl and alkenyl glycerols (Table 1, Figure S1B,C). The staining reaction was also optimized (Figure S2, Supplementary) to obtain clearly defined bands after carbonization.

**Figure 3:**
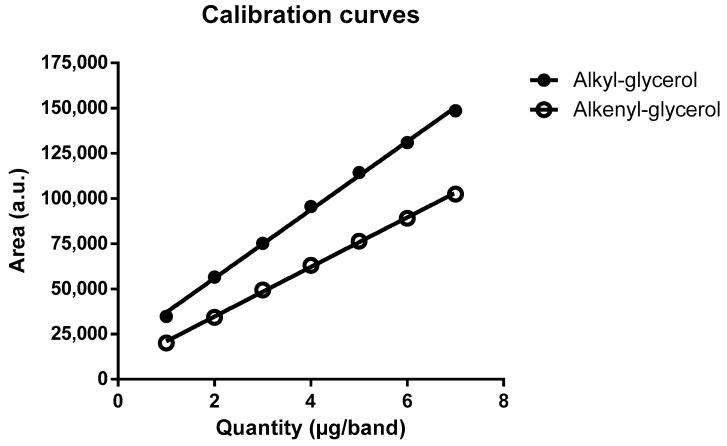
Calibration curves for the alkyl and alkenyl glycerol standards. The resulting calibration curves with linear models were built by plotting the peak area of each ether glycerol standard versus the applied quantity for amounts ranging from 1000 ng per band to 7000 ng per band, with an average of six replicates per level and per analyte. The equations of the linear models are presented as y = ax + b, were y is the area of the peak, a is the slope of the curve, x is the quantity of ether glycerol standard deposited on the plate, b is the intercept of the curve and r^2^ is the coefficient of determination of the linear model. The equation of the regression line obtained for alkyl glycerol is 19,088x + 17,600, with an r^2^ of 0.9940. The equation of the regression line obtained for alkenyl glycerol is 13,687x + 6778, with an r^2^ of 0.9932.

**Figure 4:**
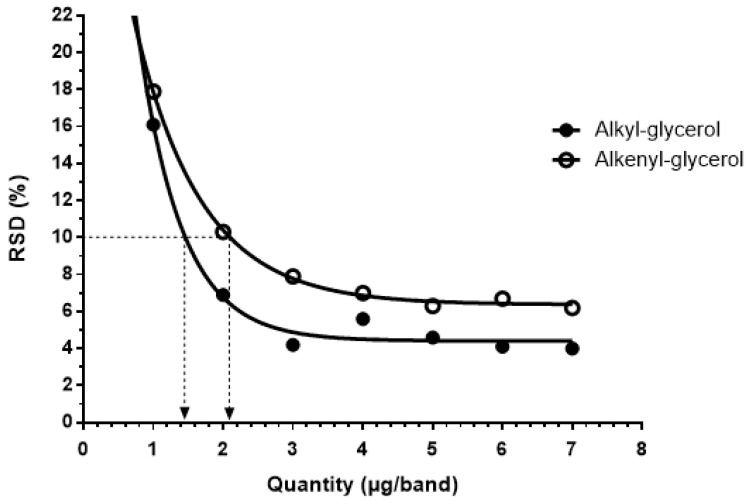
Precision profile obtained for the alkyl and alkenyl glycerol species. Relative standard deviation (RSD, %) was determined for amounts ranging from 1000 to 7000 ng/band of alkyl and alkenyl glycerol standards. Limits of quantification (LOQ) for both ether glycerol species were determined for an RSD value of 10%.

The developed method was found to be linear for amounts of ether glycerol standard ranging from 1000 to 7000 ng/band for both alkyl and alkenyl glycerol, with a coefficient of determination, r², for the regression line equations above 0.99 for both species (Figure 3). Based on the method using the standard deviation of the response and the slope of the regression line (Table 2, Method 2), the LOD and LOQ obtained for the alkyl glycerol standard are 407 and 1235 ng/band, respectively. For the alkenyl glycerol standard, the LOD and LOQ are 776 and 2352 ng/band, respectively. The results obtained for both ether glycerol species and each tested method can be found in Table 2 and Figure 4, respectively.

Concerning precision, repeatability was measured on the same day by the same operator (intraday assays), and intermediate precision was assessed over a two-month period by several operators (interday assays). RSD values under 10% were obtained for alkyl glycerol ranging from 2000 to 7000 ng/band and under 20% below 2000 ng/band. For alkenyl glycerol, the RSD was under 20% for amounts ranging from 1000 to 7000 ng/band (Table 3 and Figure 4).

These results show a satisfactory precision of the proposed method. Regarding accuracy, results from the recovery studies performed according to the method described by Pinault et al. [16] are presented in Table 4.

For both ether glycerol species, recoveries were between 96.39 and 100.84%, with an RSD under 5%, indicating a satisfactory accuracy of the proposed method [22].

Band identification was confirmed by performing acid hydrolysis (see Section 3) on both ether glycerol standards and total reduced lipids from samples (see Section 2, Application to biological samples). This method, which has been described in the literature [23,24,25], is specific to ether lipids, as it is based on their chemistry; an ether bond is stable in acid conditions, whereas a vinyl ether bond is hydrolyzed, resulting in the degradation of alkenyl glycerol species.

The alkenyl glycerol band disappeared completely after acid hydrolysis on both the alkenyl glycerol standard and the sample, whereas the alkyl glycerol band remained present on HPTLC (Figure S3A,B). This result was also verified by ^1^H NMR, where the multiplets at 4.3 and 5.9 ppm corresponding to the vinylic protons, as described in the literature [26,27] and as confirmed in our alkenyl glycerol standard, disappeared after acid hydrolysis of the standard and the sample (Figure S4).

A summary of the validation parameters can be found in Table 5.

### 2.3. Statistical Analysis of the Validation

Variance homogeneity between datasets was verified by analysis of variance (ANOVA) using Cochran’s C test, showing no significant difference of variance at a 95% confidence interval (Table 3). The fitted linear regression models were validated using Fisher’s test. For both ether glycerol species, the slopes of the models were found to be significantly different from 0, with a *p* < 0.05. Moreover, the coefficient of determination r^2^ being close to 1 in both models suggests a good fit between the experimental data and the established models.

### 2.4. Application for Biological Samples

Different types of biological samples were tested to confirm that this method is suitable for samples with a complex matrix. Oils, adipose, muscle and tumor tissues were used. 

**Figure 5:**
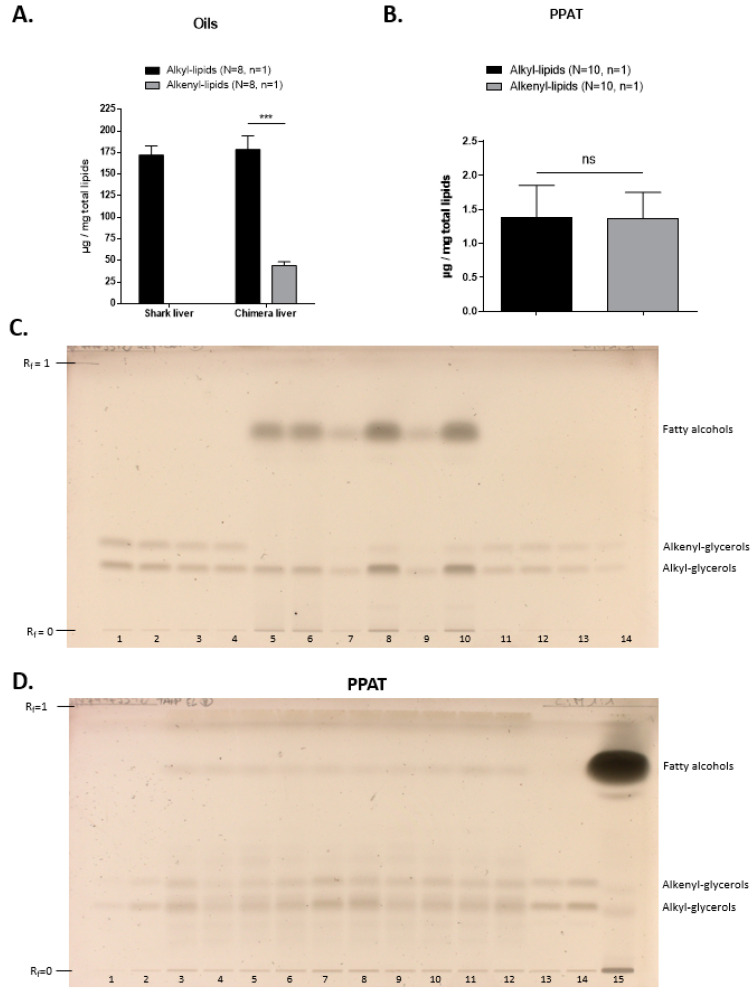
Quantification of the alkyl and alkenyl lipids in oils and PPAT with the proposed HPTLC method. The quantity of ether lipids deposited was calculated by quantifying the amount of ether glycerols by HPTLC densitometry. We next determined the total quantity of ether lipids that was contained in the whole sample. Finally, we relativized this total quantity of ether lipids to the quantity of total lipids extracted in the sample to obtain our results in µg of ether lipids per mg of total lipids. The volumes deposited can be found in Section 3. (mean ± SD, Mann–Whitney, ns: not significant, *** *p* < 0.001). (**A**) A total of eight samples of each oil were reduced and applied once on HPTLC (*n* = 8, *n* = 1). (**B**) Total lipids of PPAT samples from ten different patients were extracted, reduced, purified and applied once on HPTLC (*n* = 10, *n* = 1). (**C**) HPTLC plate for marine oils samples. The samples were deposited as follows: lanes 1 to 4 and 11 to 14: ether glycerol standards; lanes 5 and 6: reduced lipids from two shark liver oil samples, deposited once for each sample; lanes 7 to 10: reduced lipids from two chimera liver oil samples, deposited twice for each samples with different volumes to quantify either alkyl glycerols (lanes 7 and 9) or alkenyl glycerols (lanes 8 and 10). (**D**) HPTLC plate for PPAT samples. The PPAT samples from ten different patients were deposited as follows: lanes 1 to 2 and 13 to 14: ether glycerol standards; lanes 3 to 12: purified reduced lipids from PPAT samples, deposited once for each sample (*n* = 10, *n* = 1); lane 15: non-purified PPAT sample.

For analysis of adipose, muscle and tumor tissues, a purification by solid-phase extraction (SPE) was required due to lipid composition heterogeneity and the lower amount of ether lipids compared to marine oils. Purification by SPE was carried out after total lipid extraction and reduction to eliminate the fatty alcohols formed during the reduction and to obtain samples concentrated in ether glycerols (Section 3.8.1, Section 3.8.2 and Section 3.8.3). Different stationary phases were tested and are listed in Table S1. Samples before and after purification are shown in Figure 5D and Figure S5C.

The quantities of alkyl lipids and alkenyl lipids per mg of total lipids in each sample can be obtained with the quantities of alkyl and alkenyl glycerols measured by densitometric analysis on the HPTLC plates. After HPTLC analysis of the lipids extracted from oils (Figure 5A,C), we found that shark liver oil possesses around 175 µg of ether lipids per mg of total lipids, and they are present as alkyl lipids only, meaning that alkyl lipids account for less than 20% of lipids in shark liver oil. However, for chimera liver oil, we found 175 µg of alkyl lipids and 50 µg of alkenyl lipids per mg of total lipids; thus, ether lipids represent about 22% of chimera liver oil total lipids. Interestingly, we found a significantly higher amount of alkyl lipids compared to alkenyl lipids in chimera liver oil, as previously observed by Hardy and Mackie [28].

In human periprostatic adipose tissue (PPAT), tissues known to contain an important amount of lipids, less than 0.5% of lipids are ether lipids, with approximately the same amounts of alkyl lipids and alkenyl lipids: around 1.3 µg of each ether lipid species per mg of total lipids (Figure 5B,D). This low amount of ether lipids was previously observed in subcutaneous and visceral adipose tissues [3].

We also verified that our method was suitable for the analysis of heart and tumor tissues, two interesting physiopathological tissues for ether lipids [9].

In non-human primate heart (NHP), ether lipids were found to represent less than 5% of total lipids, with almost only alkenyl lipids in our samples tested (Figure S5A,C), consistent with literature reports [8]. In tumors from the MDA-MB-435s cell line, a low amount of ether lipids was found, with around 2 µg of each ether lipid species per mg of total lipids (Figure S5B,D); however, little is known about ether lipids from cell lines [29]. These results, although they do not account for a precise quantification of ether lipids in NHP heart and tumors due to the low amount of replicates, show that this method can be adapted to various matrices.

### 2.5. Use of This Method in Combination with Lipidomics

The proposed method is well designed to be used for a lipidomic study and in combination with an HPLC-MS/MS system. After total lipid extraction, a small part of the sample is readily processable by HPLC-MS/MS to identify the lipids in the sample. In addition, quantifications of other lipid subspecies can be performed by HPTLC [30] on a fraction of the total lipid extract, depending on the lipid species of interest. The remaining part of the total lipid extract can then be reduced and analyzed with our developed HPTLC method to quantify the subspecies of alkyl and alkenyl glycerolipids. The benefit of this HPTLC method is that it permits an absolute quantification of the analyzed alkyl and alkenyl glycerolipids, whereas the calibration of an HPLC-MS/MS can be difficult due to the complexity of the ether lipid species [31]. Indeed, very few ether lipid commercial standards are available, leading to an estimation only and not a rigorous total amount of alkyl and alkenyl lipids. Moreover, in the specific case of ether lipids, HPLC-MS/MS does not enable the separation of positional isomers [32], meaning that alkyl lipids with at least one unsaturation and alkenyl lipids cannot be differentiated without further treatment [23]. Finally, the combination of the two methods enables the realization of complete and thorough studies [33] so as to obtain a more precise view of the lipidome: HPTLC provides a global view of the content of alkyl and alkenyl glycerolipids in the sample, whereas HPLC-MS/MS identifies the ether lipid species, as well as the other lipids within the sample.

## 3. Materials and Methods

### 3.1. Chemicals and Reagents

1-O-Octadecyl-rac-glycerol (alkyl-glycerol) (ref B402) and 1-O-1′-(Z)-Octadecenyl-*sn*-glycerol (alkenyl-glycerol) (ref 852650P) were obtained from Sigma-Aldrich and Avanti Polar. Vitride^®^ (sodium bis(2-methoxyethoxy)aluminiumhydride) (ref 196193), hydrochloric acid (ref 524525), oleyl alcohol (fatty alcohol) (ref 369314), deuterated chloroform (ref 151823), tetramethylsilane (ref 87920) and butylated hydroxytoluene (ref W218405, BHT) were purchased from Sigma-Aldrich (Saint-Quentin, France). All solvents used were of analytical grade.

### 3.2. HPTLC Apparatus

An automatic TLC sampler 4 (ATS4, Camag, Muttenz, Switzerland) equipped with a 25 µL syringe (ref 695.0053, Camag, Muttenz, Switzerland) was used for sample application under a stream of nitrogen on 20 × 10 cm F_254_ glass plates coated with a 200 µm layer of 60 Å silica gel (Merck, Darmstadt, Germany). Plates were developed in a twin-trough glass chamber (Camag, Muttenz, Switzerland) and carbonized on a hot plate (Plate Heater III, Camag, Muttenz Switzerland). Results were obtained with a TLC visualizer I (Camag, Muttenz, Switzerland) working under winCATS Planar Chromatography Manager software (version 1.4.610, Camag, Muttenz, Switzerland), and densitometry was performed with VideoScan TLC/HPTLC software (version 1.02.00, Camag, Muttenz, Switzerland).

### 3.3. Chromatographic Conditions

Before sample application, the 20 × 10 cm glass plates pre-coated with silica gel were developed once in chloroform: methanol (1:1; *v/v*), air-dried and activated in an oven for 30 min at 110 °C. Standard and sample solutions were then applied by spray-on technique with an ATS4 sample applicator as 10 mm wide bands, 8 mm from the bottom and 15 mm from the edge of the plate. Application was performed at a rate of 150 nL/s under a stream of nitrogen at 6 bars to enable continuous drying, thus avoiding diffusion, permitting better separation and homogenous quantification. Linearly ascending development was carried out in a twin-trough glass chamber with a petroleum ether: diethyl ether: acetic acid (30:70:0.5; *v/v/v*) mobile phase prepared weekly. The glass chamber was first pre-equilibrated with the mobile phase at room temperature for 15 min without saturation paper. Following development, plates were air-dried for 120 min under a ventilated hood before being dipped for 50 s in a 7% sulfuric acid in absolute ethanol staining solution prepared daily. Plates were air-dried for another 120 min before carbonization for the staining reaction at 140 °C for 14 min. Pictures of the plates under white light illumination were obtained using a TLC visualizer, and densitometric analysis was performed with VideoScan.

### 3.4. Standard Solution Preparation

Standard solutions of alkyl glycerol and alkenyl glycerol used for the quantification of alkyl and alkenyl glycerolipids, respectively, were prepared at 1 mg/mL in chloroform: methanol (2:1; *v/v*) with 250 µM of BHT and stored at −20 °C. Alkenyl lipids are known to have very low stability against oxidation due to their *sn-1* vinyl ether bond [34]. Thus, the alkenyl glycerol standard solution must be prepared weekly despite the use of antioxidant. Alkyl lipids, however, do not share this stability issue.

### 3.5. Method Validation

The developed method for the analysis of ether lipids was validated according to the ICH guidelines [19]. Selectivity, linearity, sensitivity, accuracy, repeatability and intermediate precision were studied.

### 3.6. Statistical Tests

Statistical analyses were performed with StatPlus 6.03.2009 (AnalystSoft, Alexandria, VA, USA). Graphical representations were performed by using GraphPad Prism (version 6.01) (La Jolla, CA, USA). 

### 3.7. Applications to Biological Samples

#### 3.7.1. Non-Human Primate Heart Samples

A whole NHP heart exposed to cigarette smoke was sampled, washed with PBS and stored at −20 °C before lipid extraction. Heart tissue came from a euthanized healthy pre-pubescent (3 years old) female cynomolgus macaque (Macaca Fascicularis, Vietnam) weighing 3 kg and obtained from Bioprim^®^ (Baziege, France). The macaque was housed under conventional conditions in the animal facility (PST, Tours, France) in accordance with the latest European legislation (Directive 2010/63/UE). The animal was placed under an experimental protocol of cigarette smoke exposition conducted according to NIH guidelines for the care and use of laboratory animals. This protocol was approved by the Ethics Committee of Val de Loire Univeristé and the Ministry of Higher Education and Research (Notification: APAFIS#14748-2018041713242393 v7).

#### 3.7.2. Periprostatic Adipose Tissue Samples

PPAT was sampled during a surgical procedure in 10 prostate cancer patients treated by radical prostatectomy at Tours University Hospital. During the surgical procedures, pieces of PPAT were sampled, sent to the biological resource center and immediately frozen at −80 °C. The patients included in the study signed an informed consent (after agreement of the Ethical Committee/IRB; clinical trials registration number NCT03214315). 

#### 3.7.3. Tumor Samples

The human cancer cell line MDA-MB-435s, obtained from the American Tissue Culture Collection, was grafted in mice to obtain an orthotopic mammary-tumor model following the protocol described by Chantôme et al. [35]. Mice were bred and housed at Inserm U892 (Nantes-University) under animal care license n°44565 [35]. The primary tumors removed were stored at −80 °C until lipid extraction.

### 3.8. Lipid Extraction, Reduction and Purification

#### 3.8.1. Lipid Extraction

No lipid extraction was necessary for oils.

Lipids from PPAT, mostly neutral lipids, were extracted according to a method adapted from Folch et al. [36]. Briefly, PPAT in a solution of 5 volumes of chloroform: methanol (2:1; *v/v*) and 1 volume of aqueous sodium chloride 0.73% was first put in an ultrasonic bath (Branson 5510) at room temperature for 20 min. After centrifugation (500× *g*, 10 min at 4 °C), the organic layer containing the lipids was filtered on cotton, dried on anhydrous sodium sulfate and evaporated.

NHP heart and tumors containing a high amount of membrane lipids were extracted according to a method adapted from Bligh and Dyer [37]. A preliminary step of disruption and homogenization was performed in chloroform: methanol: water (2:4:1.6; *v/v*) on ice using an Ultra-Turrax homogenizer (T 25, IKA) at 24000 rpm for 30 s to 3 min per sample to obtain a homogenous sample. This solvent enables membrane disorganization, with the denaturation and precipitation of most proteins and the solubilization of lipids. After centrifugation, (500× *g*, 10 min at room temperature), the obtained supernatant was transferred to a second tube, whereas the pellet was extracted again in chloroform: methanol (2:4; *v/v*) to recover more lipids. Chloroform: water (2:2; *v/v*) was then added to both tubes for phase partitioning. The lower chloroform phases containing the lipids were then filtered on cotton, dried on anhydrous sodium sulfate and evaporated.

After evaporation, total lipids were weighted to proceed immediately to lipid reduction and avoid the oxidation of alkenyl lipids. The amount of tissue used and the total lipids recovered after extraction can be found in Table 6. For short-term storage, total lipids can be diluted in chloroform: methanol (2:1; *v/v*) with 250 µM of BHT, closed under nitrogen and stored at −80 °C.

#### 3.8.2. Lipid Reduction

Reduction of the ester and phosphate bonds was then carried out according to the method described by Pinault et al. [16]. Briefly, 500 µL of Vitride^®^ was added to 10 mg of extracted lipids in 5 mL of diethyl ether: n-hexane (80:20; *v/v*), closed under nitrogen and reacted for 30 min at 37 °C, with vortexing for 5 s every 10 min. The reduction was stopped on ice by slowly adding 8 mL of ethanol: water (20:80; *v/v*), obtaining ether glycerols (1-O-alkyl-glycerols and 1-O-alkenyl-glycerols, referred to as alkyl glycerols and alkenyl glycerols) and fatty alcohols (Figure 2 and Figure S6). After centrifugation (500× *g*, 10 min at 4 °C), the organic layer was filtered on cotton, dried on anhydrous sodium sulfate, evaporated and diluted in 750 µL n-hexane. The amount of total lipids used for reduction and the corresponding amount of reduced lipids recovered can be found in Table 7.

#### 3.8.3. Purification

A purification step by SPE was performed to remove impurities after reduction. The purification conditions were optimized for amounts of reduced lipids ranging from 10 to 30 mg (Table 8), depending on the expected abundance of ether lipids in the samples, and purification was performed on an SPE Chromabond SB cartridge (ref 730426, Macherey-Nagel, Hoerdt, France) using Visiprep™ SPE (ref 57044, Supelco) vacuum collectors as follows. After a preconditioning step with two times 6 mL n-hexane, the solution of reduced lipids was loaded onto the cartridge under a vacuum to enable a flow rate of one to two drops per second. Elution was carried out first with 5 mL n-hexane: dichloromethane (30:70; *v/v*; first fraction containing fatty alcohols), followed by 5 mL n-hexane: dichloromethane (20:80; *v/v*; second fraction) and then 6 mL dichloromethane: diethyl ether (50:50; *v/v*) (third fraction containing reduced ether lipid species) and, in the same tube, 6 mL diethyl ether: ethyl acetate (80:20; *v/v*). Finally, the third fraction was concentrated under vacuum and solubilized in chloroform: methanol (2:1; *v/v*) before analysis by HPTLC. The solubilization and deposited volumes can be found in Table 8.

Biological samples were quantified by HPTLC immediately after extraction and purification to avoid oxidation of the alkenyl lipid species.

### 3.9. Acid Hydrolysis

Band identity confirmation of the alkyl and alkenyl glycerols was verified by first performing acid hydrolysis, a method specific to ether lipids adapted from the literature [23,24,25]. Acidic conditions hydrolyze all alkenyl lipid species by reacting on the vinyl ether bond and forming fatty aldehydes but not reacting on ether bonds. Thus, performing acid hydrolysis on the total lipid extract is a simple way to verify the specificity of the HPTLC method. Alkyl glycerols should remain in the same quantity, and the band for alkenyl glycerols should totally disappear. Briefly, five drops of 12 M hydrochloric acid were added to 3 mg of dried total reduced lipids or 0.5 µg of ether glycerol standard. After 5 min, 600 µL of n-hexane: isopropanol (3:2; *v/v*) and 150 µL of water were added to extract the hydrolyzed reduced lipids. The organic phase was recovered and evaporated. The samples before and after acid hydrolysis can be analyzed both on HPTLC and in ^1^H NMR to verify the disappearance of the alkenyl glycerol band after hydrolysis on HPTLC and the disappearance of the multiplets corresponding to the chemical shift for both vinylic protons in ^1^H NMR.

### 3.10. ^1^H NMR

^1^H NMR spectra were obtained on a 300MHz spectrometer (Bruker Avance, 7.05 Tesla) in deuterated chloroform (CDCl_3_) with 0.03% tetramethylsilane commercial standard of 1-O-1′-(Z)-Octadecenyl-*sn*-glycerol (alkenyl-glycerol): ^1^H NMR (CDCl_3_) *δ* 5.97 (dt, *J* = 6.24 Hz, *J* = 1.32 Hz, 1H_vinyl-ether_), 4.29 (m, 1H_vinyl-ether_), 3.73 (m, 3H_glycerol_), 3.52 (m, 2H_glycerol_), 2.02 (m, 2H_α vinyl-ether_), 1.26 (m, 28H), 0.87 (t, *J* = 6.50 Hz, 3H_CH3_).

## 4. Conclusions

We developed a simple HPTLC method for the quantification of total alkyl and alkenyl glycerolipids thanks to the separation of alkyl and alkenyl glycerol. This method was successfully used to quantify alkyl glycerolipids and alkenyl glycerolipids in oils and adipose tissues. We showed that it could also be applied to matrices, such as muscle and tumor tissues. It should be noted that a sufficient amount of lipids is required, especially for samples containing a low amount of ether lipids.

## Figures and Tables

**Table 1:** Obtained HPTLC profile.

Ether Glycerol Classes	Petroleum Ether: Diethyl Ether: Acetic Acid (30:70:0.5; *v/v/v*)		
	R_f_ Start	R_f_ Max	R_f_ End
Alkyl glycerol	0.294	0.337	0.379
Alkenyl glycerol	0.404	0.444	0.485

The mean retention factors (R_f_) of the start of the peak (R_f_ start), the maximum intensity of the peak (R_f_ max) and the end of the peak (R_f_ end) were estimated at six different quantities of alkyl and alkenyl glycerol standards in duplicates on one HPTLC plate.

**Table 2:** Sensitivity of the proposed HPTLC method assessed by three different methods.

Ether Glycerol Classes	Method 1 ^a^		Method 2 ^b^		Method 3 ^c^	
	LOD (ng/band)	LOQ (ng/band)	LOD (ng/band)	LOQ (ng/band)	LOD (ng/band)	LOQ (ng/band)
Alkyl glycerol	436	1459	407	1235	/	~1450
Alkenyl glycerol	716	2388	776	2352	/	~2100

^a^ Visual method based on the visual assessment of the signal-to-noise ratio (S/N) observed with low quantities of analytes by calculating LOD = 3 × (S/N) and LOQ = 10 × (S/N), calculated with an average of 43 replicates. ^b^ Based on the standard deviation of the response (SD) and the slope (S) of the calibration curve by calculating LOD = 3.3 × (SD/S) and LOQ = 10 × (SD/S), with an average of 13 replicates. ^c^ Based on the Eurachem approach [21] which evaluates the LOQ as the quantity of analyte corresponding to an RSD of 10% (see Figure 4).

**Table 3:** Precision of the proposed HPTLC method.

Ether Glycerol Classes	Quantity (ng/band)	Repeatability (%)	Intermediate Precision (%)	LOL (ng/band)	Cochran	ANOVA Validation Fisher’s Test
					Ccalc < Cref	
					(*p* < 0.01)	
Alkyl glycerol	1000	12.4	16.1	7000	0.3192 < 0.397	Accepted
	2000	7.1	6.9			
	3000	6.4	--			
	4000	6.1	5.6			
	6000	3.8	4.1			
	7000	3.6	4.0			
Alkenyl glycerol	1000	18.6	17.9	7000	0.3387 < 0.418	Accepted
	2000	17.3	10.3			
	3000	15.2	7.9			
	4000	6.5	7			
	6000	5.9	6.7			
	7000	5.7	6.2			

The repeatability and the intermediate precision of the proposed method for both ether glycerol species were estimated at six different quantities of standards, with an average of six replicates. Results are shown as the relative standard deviation (RSD) of the mean of the signal in %.

**Table 4:** Accuracy of the proposed HPTLC method.

Ether Glycerol Classes	Amount of Ether Glycerol Spotted (µg/band)	Percentage of Ether Glycerol Added (%)	Theoretical Amount (µg/band)	Amount Determined (Mean ± SD) (µg/band)	% Recovery (Mean ± SD)
Alkyl glycerol	2.00	50	3.00	2.92 ± 0.08	97.17 ± 2.56
	2.00	100	4.00	3.98 ± 0.06	99.62 ± 1.44
	2.00	150	5.00	5.02 ± 0.07	100.66 ± 1.84
Alkenyl glycerol	2.00	50	3.00	2.89 ± 0.01	96.39 ± 0.26
	2.00	100	4.00	3.95 ± 0.13	98.68 ± 3.21
	2.00	150	5.00	5.04 ± 0.14	100.84 ± 2.90

The results of the recovery studies were estimated at 50, 100 and 150% of each ether glycerol species added to the 2.00 µg initial quantity deposited. The percentages of recovery obtained are shown as mean ± standard deviation, with three replicates.

**Table 5:** Validation parameters of the proposed HPTLC method.

Ether Glycerol Classes	Alkyl Glycerol	Alkenyl Glycerol
Specificity	Yes	Yes
Linearity range (ng/band) ^a^	1000–7000	1000–7000
Linear regression equation	19,088x + 17,600	13,687x + 6778
Slope ± SD	19,088 ± 3199	13,687 ± 3294
Regression coefficient (r^2^) *±* SD	0.9940 ± 0.0025	0.9932 ± 0.0044
Limit of detection (ng/band) ^b^	407	776
Limit of quantification (ng/band) ^b^	1235	2352
Repeatability (%) ^a^	3.6–12.4	5.7–18.6
Intermediate precision (%) ^a^	4.0–16.1	6.2–17.9

The amounts of alkyl and alkenyl glycerol were calculated in ng per band. ^a^ Average of six replicates at each level. ^b^ Calculated based on the standard deviation of the response (SD) and the slope (S) of the calibration curve, with an average of 13 replicates.

**Table 6:** Amount of tissue and corresponding total lipids extracted.

Tissue	Tissue Weight (mg) *Median [Min–Max]*	Total Lipids Weight (mg) *Median [Min–Max]*
Chimera liver oil	/	31.3 *[28.9–34.0]*
Shark liver oil	/	31.7 *[29.5–34.6]*
PPAT	966.8 *[356.7–1679.0]*	580.2 *[182.0–1241.9]*
Non-Human Primate Heart	637.6 *[565.5–2443.1]*	27.6 *[23.6–36.6]*
Tumors	335.4 *[150.7–440.0]*	15.2 *[3.4–23.7]*

**Table 7:** Amount of total lipids used for reduction and corresponding reduced lipids.

Tissue	Lipids Weight for Reduction/Vitride (mg) *Median [Min–Max]*	Reduced Lipids Weight (mg) *Median [Min–Max]*
Chimera liver oil	31.3 *[28.9–34.0]*	32.5 *[29.3–35.4]*
Shark liver oil	31.7 *[29.5–34.6]*	30.3 *[28.2–34.2]*
PPAT	36.1 *[29.3–40.0]*	31.3 *[24.2–35.2]*
Non-Human Primate Heart	11.7 *[7.7–36.6]*	7.8 *[6.8–28.3]*
Tumors	16.0 *[11.1–23.7]*	13.0 *[12.9–19.3]*

**Table 8:** Amount of reduced lipids purified and their corresponding solubilization and deposited volumes.

Tissue	Lipids Weight for Purification/SPE (mg) *Median [Min–Max]*	Solubilization Volume (mL) *Median [Min–Max]*	Volume Deposited (µL)
Chimera liver oil	/	10.5 *[9.4–11.4]*	for alkyl glycerols: 5 for alkenyl glycerols: 20
Shark liver oil	/	10.8 *[10.0–11.8]*	10
PPAT	31.3 *[24.2–35.2]*	0.20	20
Non-Human Primate Heart	7.8 *[6.8–28.3]*	0.78	for alkyl glycerols: 30 for alkenyl glycerols: 15
Tumors	13.0 *[12.9–19.3]*	0.50	40

## Supplementary Materials

The following supporting information can be downloaded at: https://www.mdpi.com/article/10.3390/md20040270/s1, Figures S1–S6: optimization of the method, results for muscle and tumor matrices and Vitride^®^ reduction; Table S1: optimization of the purification.
