# Supplementary material for: Development of a High-Performance Thin-Layer Chromatography Method for the Quantification of Alkyl Glycerolipids and Alkenyl Glycerolipids from Shark and Chimera Oils and Tissues
Source: Mar Drugs. 2022 Apr 18;20(4):270. doi: 10.3390/md20040270 (PMC9029064; doi:10.3390/md20040270)
Supplement: Supplementary file 1 [file marinedrugs-20-00270-s001.zip › marinedrugs-1692069-supplementary.pdf]

Supplementary Materials:

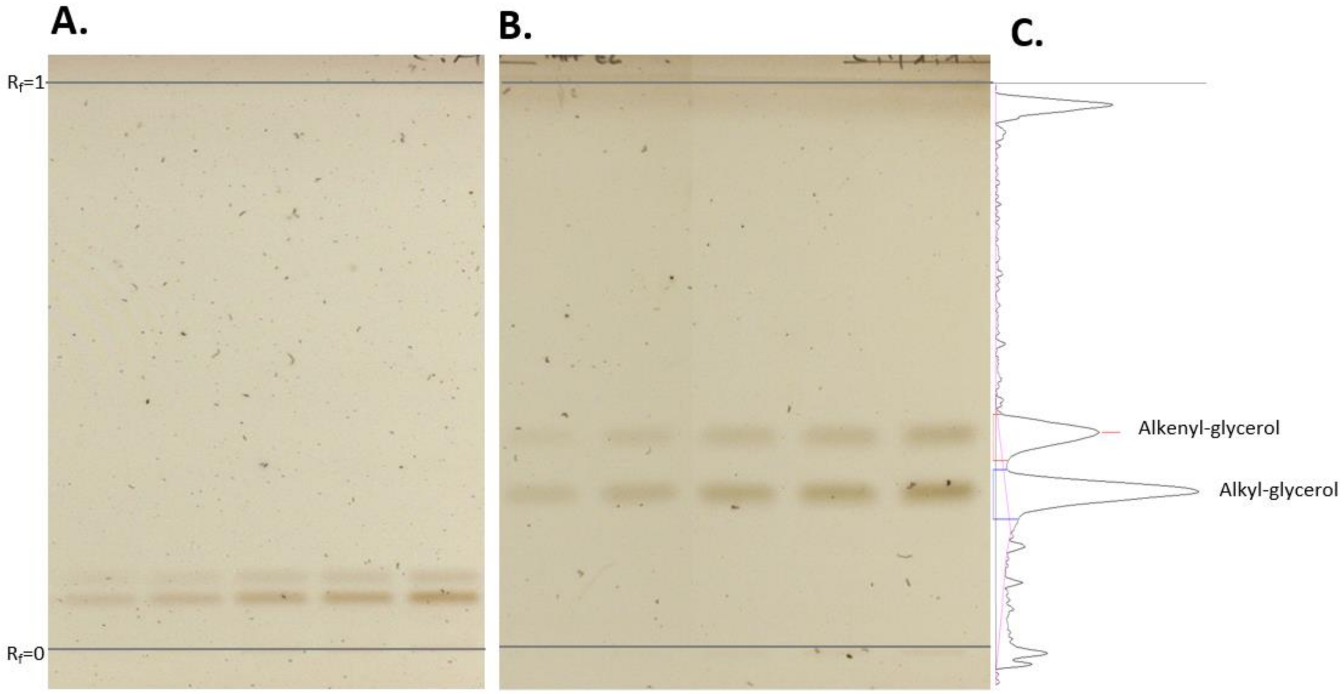

Figure S1. Optimization of the mobile phase. 2 to 6 µg of each alkyl- and alkenyl-glycerol standard were co-deposited from left to right at R_f=0 and eluted in different mobile phases until R_f=1. The carbonization was the same for both plates. A. Elution was carried out in petroleum ether : diethyl ether : acetic acid (60:40:1; v/v/v). Alkyl- and alkenyl-glycerol standards are only slightly separated. B. Elution was carried out in petroleum ether : diethyl ether : acetic acid (30:70:0.5; v/v/v). Alkyl- and alkenyl-glycerol standards are clearly separated. C. Densitometric profile obtained for the HPTLC plate shown in B, 5th lane (5 µg/band)

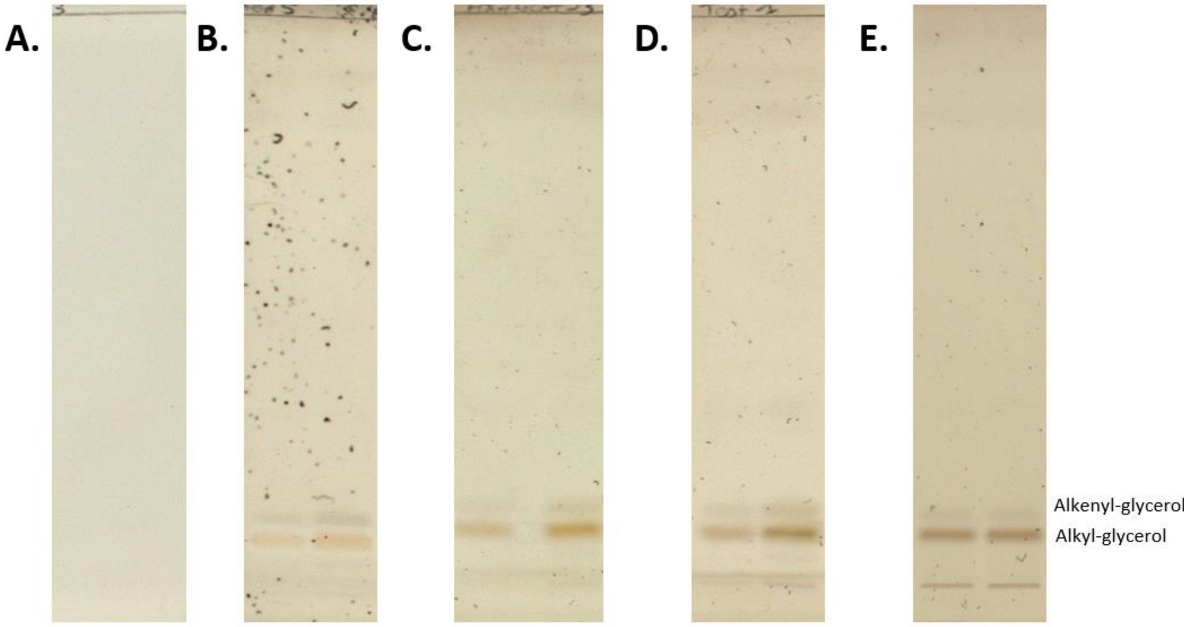

Figure S2. Optimization of the staining and carbonization reactions. The staining conditions tested on parts of HPTLC plates for 5 and 6 µg of alkyl- and alkenyl-glycerol standard per band were the following: A. Staining by 30 seconds dipping in acetic acid : sulfuric acid : absolute ethanol (20:1:170, v/v/v), carbonization for 10 minutes at 130°C. No carbonization of the alkyl- and alkenyl-glycerol occurred. B. Staining by 30 seconds dipping in 25% sulfuric acid in absolute ethanol, carbonization for 5 minutes at 130°C. The plate was too damaged after carbonization to enable the densitometric analysis. C. Staining by 30 seconds dipping in 5% sulfuric acid in absolute ethanol, carbonization for 11 minutes and 30 seconds at 130°C. The carbonization was incomplete resulting in a bias of quantification. D. Staining by 30 seconds dipping in 8% sulfuric acid in absolute ethanol, carbonization for 10 minutes at 130°C. The resulting bands of alkyl- and alkenyl-glycerol were too diffuse for a precise quantification. E. Staining by 50 seconds dipping in 7% sulfuric acid in absolute ethanol, carbonization for 14 minutes at 140°C. These were the best conditions found.

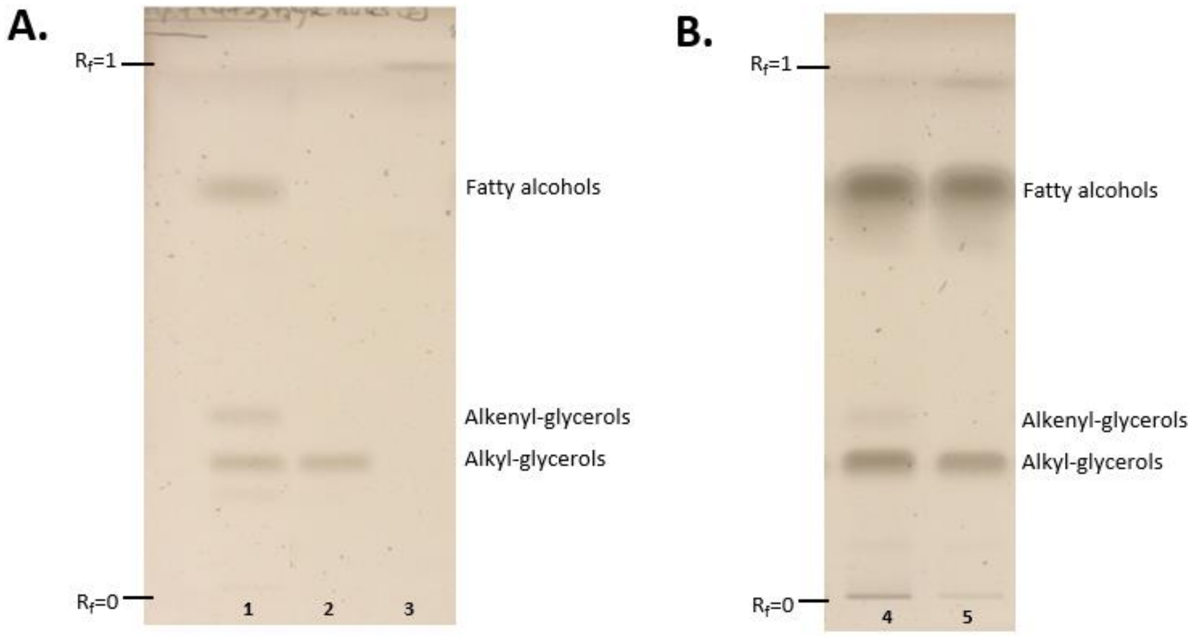

Figure S3. Confirmation of the identity of the alkenyl-glycerol species by HPTLC after acid hydrolysis of the ether-glycerol standards and the chimera liver oil sample. A. 5 µg of alkyl- and alkenyl-glycerol standards were deposited before and after acid hydrolysis as follows: lane 1: alkyl- and alkenyl-glycerol co-deposited before acid hydrolysis with 5µg of fatty alcohol standard; lane 2: alkyl-glycerol standard after acid hydrolysis; lane 3: alkenyl-glycerol standard after acid hydrolysis. B. Total reduced lipids of chimera liver oil were deposited before (lane 4) and after (lane 5) acid hydrolysis.

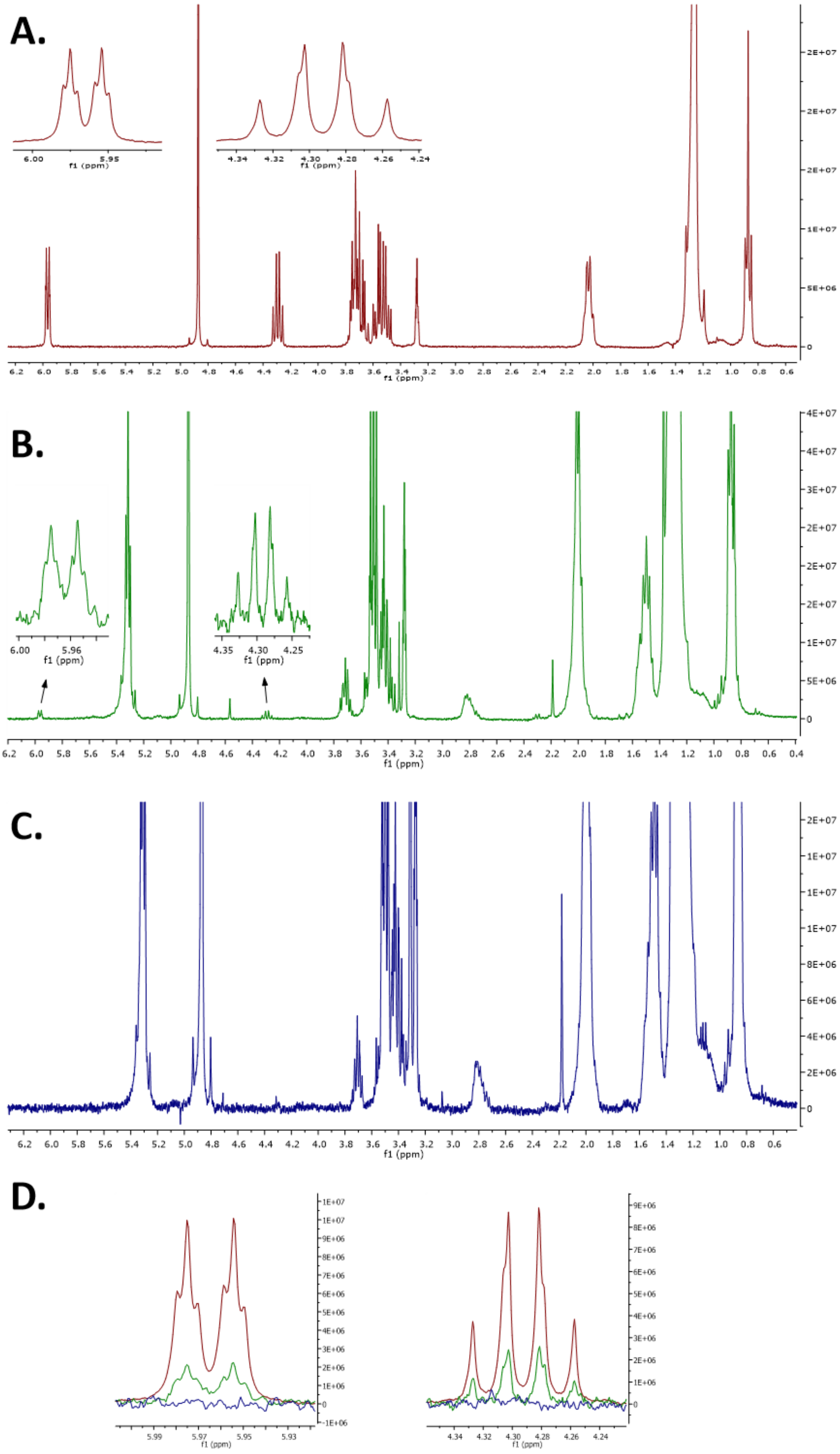

A.
B.
C.
D.
f1 (ppm)

Figure S4. Confirmation of the identity of the alkenyl glycerol species by ^{1}H NMR after acid hydrolysis of the alkenyl glycerol standard and the chimera liver oil sample. ^{1}H NMR spectra of the commercial standard of alkenyl glycerol (**A**., red) and reduced lipids from chimera liver oil sample before (**B**., green) and after (**C**., blue) acid hydrolysis. The regions at 4.3 and 5.9 ppm showing the multiplets corresponding to the vinylic protons have been expanded and overlaid (**D**.) to highlight the disappearance of the vinylic multiplets after acid hydrolysis.

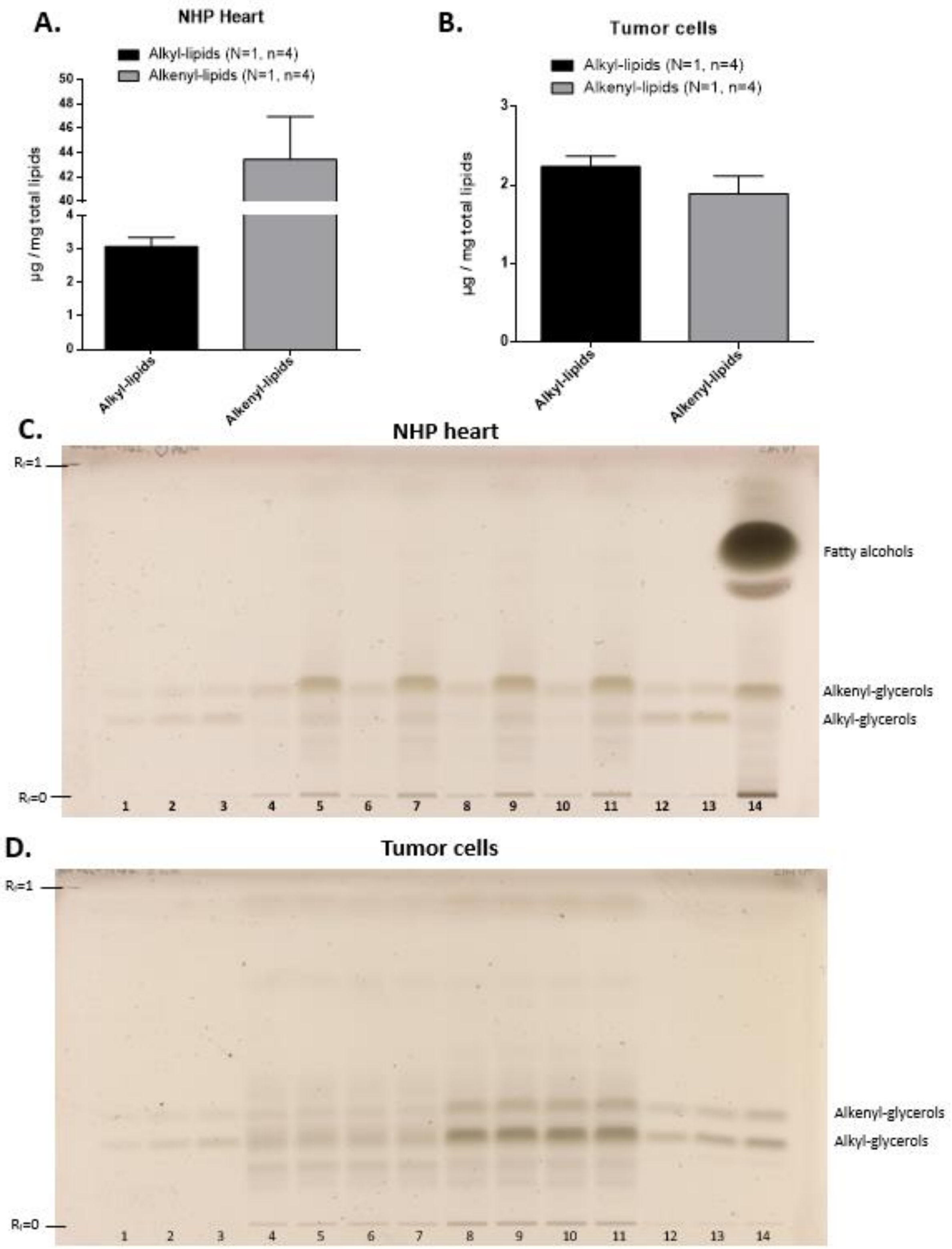

Figure S5. Results observed with the proposed HPTLC method applied to NHP heart muscle and tumor tissues. The quantity of ether-lipids was calculated by quantifying the amount of ether-glycerols by HPTLC-densitometry and relativized to the total quantity of lipids in the sample. The results are given in µg of ether-lipids per mg of total lipids and shown as mean ± SD. The quantification is possible in matrixes such as: A. NHP heart tissues, and B. tumor tissues. For each sample type, one sample of total lipids has been reduced, purified and applied four times on HPTLC (N=1, n=4). C. HPTLC plate for

NHP heart samples. The samples were deposited as follows: lanes 1 to 3 and 12 to 13: ether-glycerol standards; lanes 4 to 11: purified reduced lipids from one NHP sample deposited four different times in two different amounts to quantify alkyl-lipids (on lanes 5, 7, 9 and 11) and alkenyl-lipids (on lanes 4, 6, 8 and 10) (N=1, n=4); lane 14: non purified NHP sample. D. HPTLC plate for tumor samples. The samples were deposited as follows: lanes 1 to 3 and 12 to 14: ether-glycerol standards; lane 4 to 7: purified reduced lipids from one tumor sample deposited 4 times (N=1, n=4); lane 8 to 12: the results are above the limit of linearity and were not taken into account.

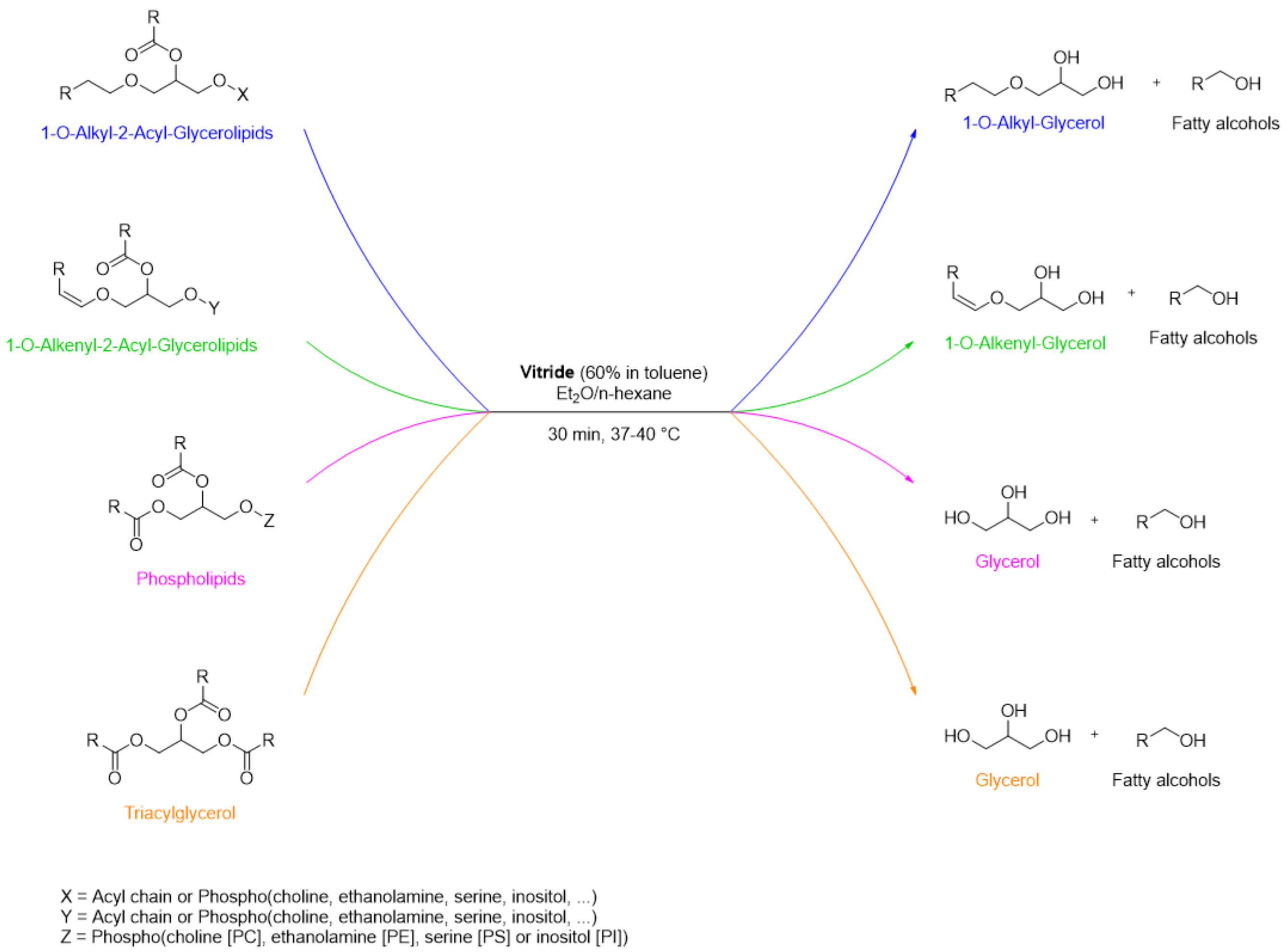

Figure S6. Reduction with Vitride® of different lipids. Vitride® reduces ester and phosphate but not ether bonds. Thus ether-glycerophospholipids (1-O-Alkyl-2-Acyl-Glycerolipids and 1-O-Alkenyl-2-Acyl-Glycerolipids) are reduced to ether-glycerols (1-O-Alkyl-Glycerol and 1-O-alkenyl-Glycerol). Phospholipids bearing no ether bond and triacylglycerols are fully reduced to glycerols. Fatty alcohols are formed. Et_2O, diethyl-ether.

Table S1 : SPE columns tested.

SPE column identification	Surface chemistry and specificities	Reference (Macherey-Nagel)	Results
Chromabond SiOH, 45 µm, 6mL/500mg	Unmodified silica gel (SiOH) – normal phase	730070	Bad separation

Chromabond HILIC, 45 µm, 6mL/500mg	Ammonium and sulfonic acid modified silica, zwitterionic, polar gel – normal phase	730594	Excellent separation, alkenyl-lipids degradation
Chromabond OH (Diol), 45 µm, 6mL/500mg	Dihydroxypropyle (Diol, OH) modified silica gel – normal phase	730418	Bad separation
Chromabond NH2, 45 µm, 6mL/500mg	Aminopropyl (NH2) modified silica phase – normal phase	730180	Bad separation
Chromabond C18 ec, 45 µm, 6mL/500mg	Octadecyl (ODS, C18 ec, RP18 ec) modified silica phase, endcapped – reversed phase	730014	Bad separation
Chromabond HLB, 45 µm, 6mL/500mg	Hydrophilic-lipophilic balanced (HLB) N-vinylpyrrolidone-divinylbenzene copolymer – reversed phase	730927	Bad separation
Chromabond SB, 45 µm, 6mL/500mg	Silica gel with quaternary ammonium modification, strongly basic anion exchanger (SAX) – ion exchange	730426	Good separation

The different stationary phases tested for the solid-phase extraction of the biological samples, their characteristics and the results obtained are listed.
